# Equilibrating SSC guidelines with individualized care

**DOI:** 10.1186/s13054-021-03813-0

**Published:** 2021-11-17

**Authors:** Jean-Louis Vincent, Mervyn Singer, Sharon Einav, Rui Moreno, Julia Wendon, Jean-Louis Teboul, Jan Bakker, Glenn Hernandez, Djillali Annane, Angélique M. E. de Man, Xavier Monnet, V. Marco Ranieri, Olfa Hamzaoui, Jukka Takala, Nicole Juffermans, Jean-Daniel Chiche, Sheila N. Myatra, Daniel De Backer

**Affiliations:** 1 Department of Intensive Care, Erasme Hospital, Université Libre de Bruxelles, 1070 Brussels, Belgium; 2 Bloomsbury Institute of Intensive Care Medicine, Division of Medicine, University College London, London, WC1E 6BT UK; 3 General Intensive Care Unit, Shaare Zedek Medical Centre, and Hebrew University Faculty of Medicine, Jerusalem, Israel; 4 Hospital de São José, Centro Hospitalar Universitário de Lisboa Central, Faculdade de Ciências Médicas de Lisboa, Nova Médical School, Lisbon, Portugal; 5 Department of Critical Care, Liver Sciences, Department of Inflammation Biology, King’s College London, London, SE5 9RS UK; 6 Service de Médecine Intensive-Réanimation, Hôpital de Bicêtre, GHU APHP, Inserm UMR S_999, Université Paris-Saclay, 94270 Le Kremlin-Bicêtre, France; 7 Division of Pulmonary, Critical Care, and Sleep Medicine, New York University School of Medicine, New York, NY 10016 USA; 8 Division of Pulmonary, Allergy, and Critical Care Medicine, Columbia University College of Physicians and Surgeons, New York, NY 10032 USA; 9 Department of Intensive Care Adults, Erasmus MC University Medical Center, 3015 GD Rotterdam, The Netherlands; 10 Department of Intensive Care, Pontificia Universidad Católica de Chile, Santiago, Chile; 11 Departmento de Medicina Intensiva, Facultad de Medicina, Pontificia Universidad Católica de Chile, Santiago, Chile; 12 School of Medicine Simone Veil, Raymond Poincaré Hospital (APHP), Department of Intensive Care Medicine, FHU SEPSIS, University of Versailles-University Paris Saclay, 92380 Garches, France; 13 Department of Intensive Care Medicine, Research VUmc Intensive Care (REVIVE), Amsterdam Cardiovascular Science (ACS), Amsterdam Infection and Immunity Institute (AI&II), Amsterdam Medical Data Science (AMDS), Amsterdam UMC, Location VUmc, Vrije Universiteit Amsterdam, 1081 HV Amsterdam, The Netherlands; 14 AP-HP, Service de médecine intensive-réanimation, Hôpital de Bicêtre, DMU CORREVE, Inserm UMR S_999, FHU SEPSIS, Groupe de Recherche Clinique CARMAS, Université Paris-Saclay, 94270 Le Kremlin-Bicêtre, France; 15 Department of Emergency and Intensive Care Medicine, Policlinico di Sant’Orsola, Alma Mater Studiorum University of Bologna, 40138 Bologna, Italy; 16 Service de Réanimation Polyvalente, Hôpital Antoine Béclère, AP-HP, Université Paris-Saclay, 92140 Clamart, France; 17 Department of Intensive Care Medicine, Inselspital, Bern University Hospital, University of Bern, 3010 Bern, Switzerland; 18 Laboratory of Experimental Intensive Care and Anaesthesiology, Amsterdam University Medical Centre, Location Academic Medical Centre, 1105 AZ Amsterdam, The Netherlands; 19 Department of Intensive Care, OLVG Hospital, Amsterdam, The Netherlands; 20 Department of Intensive Care Medicine, Centre Hospitalier Universitaire Vaudois, 1011 Lausanne, Switzerland; 21 Department of Anaesthesiology, Critical Care and Pain, Tata Memorial Hospital, Homi Bhabha National Institute, 400012 Mumbai, India; 22 Department of Intensive Care, CHIREC Hospital, Université Libre de Bruxelles, Brussels, Belgium

Sepsis is a major cause of death worldwide, not least because complex interventions need to be provided within a short window of opportunity. Evidence-based guidelines for the treatment of sepsis are therefore welcome, providing a common ground for all clinicians involved in decision-making regardless of their expertise. Such guidelines should therefore serve as an overarching reference document. As previously stated, ‘*Guidelines are the product of an explicit, systematic approach to the evaluation and synthesis of available information on a particular clinical topic. They are not a compilation of truths, but are a summary of what is accepted by the authors as the best available evidence at that time*’ [1].

As the evidence base evolves over time, the new Surviving Sepsis Campaign (SSC) guidelines [2] are timely and important. We appreciate the tremendous amount of time invested by the experts who formulated the new guidelines to provide the intensive care community with a clear and comprehensive manuscript. We also recognize the challenge of providing evidence-based guidelines when the strength of evidence available to direct recommendations is limited. Indeed, the large majority of randomized, controlled trials (RCTs) performed over the last three decades in intensive care medicine, including those in sepsis, have shown no significant beneficial effect of the tested intervention on outcomes [3]. At face value, this may simply suggest that the myriad of interventions that have been tested are all ineffective. However, it is more likely that subsets of patients who benefit from specific treatments have yet to be identified. The often broad patient inclusion criteria could easily lead to dilution of positive findings by non-responders, or to positive effects in some patients being offset by harm in others [4]. This treatment effect heterogeneity has been clearly indicated in many trials (for example [5–8]).

The most severely ill patients usually suffer less from a single condition than from a complex physiological imbalance that defies specific disease definitions. Management that strictly adheres to guidelines may not necessarily be the best option. However, personalizing management of these patients mandates that the treating clinician appreciates the clinical implications of the underlying disease(s) and individual host factors (chronic health status, physiology, and physiological reserves). Additionally, the individual response to interventions must be closely monitored and, depending on that response and whether or not the pre-set goal of that intervention has been achieved, the clinician can then decide to maintain or alter the intervention accordingly. We fully acknowledge this is demanding in resources, time, personnel, and bedside expertise. It is more convenient and less labor-*intensive* to adopt a generalized approach, yet customization is surely the underlying foundation of *intensive* care. When we forget the fundamental importance of individualized management, the ultimate result is that few interventions improve meaningful patient outcomes, especially mortality [3]. We must acknowledge and embrace natural (patho) physiological variability; sepsis is no exception to this rule. Sepsis encompasses a huge spectrum of clinical situations in terms of the type of patient involved, the clinical presentation and the response to treatment. This variability should serve to direct clinical management, enabling the clinician to adapt “recommended” care according to the specific needs of that patient. As David Sackett, the father of evidence-based medicine (EBM) stated, *“Good doctors use both individual clinical expertise and the best available external evidence, and neither alone is enough. Without clinical expertise, practice risks becoming tyrannised by evidence, for even excellent external evidence may be inapplicable to or inappropriate for an individual patient. Without current best evidence, practice risks becoming rapidly out-of-date, to the detriment of patients.”* [9]*.*

The conundrum is how to encourage guideline implementation yet at the same time promote personalized medicine. The new guidelines [2] offer little leeway for adapting the recommendations to the idiosyncrasies of each and every patient. Many of the recommendations attempt to fit most (if not all) of our patients. Sets of recommendations have been translated into bundles of care to be applied within a rigid time-frame to the “average” patient. This approach has sacrificed precision for homogenization and expediency. Some allowance for breaking the “one size fits all” guideline mold that has taken root in the last two decades would have been a daring but welcome and timely change.

We have therefore taken the liberty of putting forward some proposals of how guidelines could be adapted to individualize care (Table 1). We fully acknowledge that these recommendations are primarily based on pathophysiological considerations and clinical experience rather than on RCT data.

**Table 1:** Twenty recommendations to individualize interventions in the early resuscitation of patients with sepsis

1.	We recommend individualizing the timing of ICU admission. It should ideally be within minutes in severely ill patients but can be less urgent in less severe cases. No time limit is applicable for *all* patients. The decision may be influenced by the level of care available within ward areas and by ICU bed availability and, of course, by the physiological status and reserve of the patient
2.	We recommend individualizing the decision to admit to the ICU. Many patients develop sepsis at the end of their life. Patients with palliative care orders and treatment escalation plans that preclude advanced organ support should generally not be admitted
3.	We recommend individualizing the timing of antibiotic therapy. Administration should be prompt in the presence of septic shock but less urgent in less severe cases, enabling more time to perform investigations, confirm the diagnosis and likely source, and seek expert advice
4.	We recommend individualizing the need for and timing of tracheal intubation, based on careful clinical assessment, including level of consciousness, respiratory rate and work of breathing, hemodynamic status, and assessment of gas exchange. Delaying tracheal intubation may lead to respiratory and even cardiac arrest, with dire consequences, yet premature use of invasive mechanical ventilation can expose the patient to ventilator-induced lung injury, distant organ complications, and increased risk of nosocomial lung infection
5.	We recommend individualizing respiratory settings in mechanically ventilated patients, including driving pressure, tidal volume and level of positive end-expiratory pressure (PEEP), aiming at the lowest possible mechanical power. PEEP could be adjusted to lung recruitment capacity
6.	We recommend individualizing oxygenation targets, taking oxygen delivery into account. Exposure to high PaO_2_ levels may be associated with worse outcomes, except perhaps in necrotizing infections. Extreme oxygenation values (too conservative or too liberal) should generally be avoided
7.	We recommend individualizing sedation therapies, recognizing that many septic patients need little or even no sedation. Tracheal intubation per se is not a sufficient indication for administration of sedative agents. Sedative agents reduce vascular tone and myocardial contractility, and may also alter immune function
8.	We recommend individualizing initial fluid resuscitation. No single formula can be applied to all patients, as fluid requirements vary substantially (depending on the source of sepsis and preexisting cardiovascular function). This is particularly true for the suggestion to give at least 30 mL/kg of fluid within the first 3 h. A young patient without comorbidities is more likely to tolerate administration of a large volume of fluid than a fragile elderly patient with severe cardiac or renal disease
9.	We recommend individualizing fluid therapy using dynamic challenges. Assessment of pulse pressure variation (PPV) or stroke volume variation (SVV) is possible only in deeply sedated mechanically ventilated patients with no spontaneous breathing. Alternative methods, including fluid challenges or passive leg raising, are therefore more widely applicable
10.	We recommend individualizing the type of intravenous fluid administered. For example, albumin administration may be considered in an edematous patient with profound hypoalbuminemia or prolonged non-response to crystalloids
11.	We recommend monitoring of chloride levels if saline solutions are administered. Saline solutions should not be banned, but one must keep in mind that liberal administration of saline results in hyperchloremia, and this may result in a worsening metabolic acidosis and renal impairment
12.	We recommend individualizing the initiation of vasopressor therapy. Fluid *pre-*loading may be considered in less severe cases, whereas fluid *co*-loading parallel to vasopressor initiation should be preferred in cases of life-threatening hypotension or a low diastolic arterial pressure
13.	We recommend individualizing arterial blood pressure levels. Although a mean value of 65 mmHg may be recommended as an initial goal, the optimal level may be higher in patients with a history of hypertension, atherosclerosis or chronic kidney disease. Conversely it may be lower in younger patients without previous vascular problems, in those with chronically low arterial pressure, or in whom adequate tissue perfusion is maintained
14.	We recommend optimizing oxygen delivery, based on clinical assessment complemented by careful hemodynamic assessment including measurement of mixed (or central) venous oxygen saturation (SvO_2_) and even carbon dioxide-derived variables. A low SvO_2_ in the presence of a normal SaO_2_ indicates inadequate overall oxygen delivery to the tissues. More importantly, a normal or high SvO_2_ does not exclude tissue hypoxia
15.	We recommend a multimodal approach to assessing tissue perfusion, including mental status, urine output, peripheral perfusion, and blood lactate levels, taking into consideration the physiological reserve of the patient
16.	We recommend individualizing blood transfusion. Transfusion should be based not only on measurements of hemoglobin concentration, but on clinical evaluation including persisting signs of tissue hypoperfusion, and measurements of SvO_2_ and lactate
17.	We recommend individualizing administration of inotropic agents when tissue hypoperfusion relates to impaired cardiac function (documented at least by echocardiography). The choice and the dose of the inotropic agent should be based on individual hemodynamic monitoring with repeated measurements
18.	We recommend individualizing the decision to administer corticosteroids, not only for septic shock, but also for other conditions such as severe pneumonia and ARDS
19.	We recommend involving senior colleagues and consultants, especially since guidelines are most useful for non-experts. Team work, communication and multidisciplinary teams are essential aspects. One of the most overarching recommendations is to seek for guidance from other colleagues and to clearly document the rationale for an intervention –be it recommended or not in the guidelines
20.	We recommend carefully measuring and monitoring the effects of any therapeutic measures undertaken and deciding whether or not to continue or adjust treatment accordingly

The way forward is to be bold enough to question how we can do better. It may be time to move from a mass approach, based on pragmatic studies performed on heterogeneous populations, to tailored studies allowing both dissection and integration of the information collected in specific sepsis phenotypes. We need to embrace theragnostic approaches, using biomarkers to identify only those patients for whom the intervention is suited, and to titrate dose and duration to optimal effect. Crucially, we need to restore the application of physiology and biochemistry to the forefront of our clinical practice. Returning to Sackett: “*Evidence based medicine is not "cookbook" medicine. (…) External clinical evidence can inform, but can never replace, individual clinical expertise, and it is this expertise that decides whether the external evidence applies to the individual patient at all and, if so, how it should be integrated into a clinical decision*.” [9]. Sepsis is clearly one instance where “*one size does not fit all”*.

## Data Availability

Not applicable.
